# High Level of Uromodulin Increases the Risk of Hypertension: A Mendelian Randomization Study

**DOI:** 10.3389/fcvm.2021.736001

**Published:** 2021-09-01

**Authors:** Ruilian You, Lanlan Chen, Lubin Xu, Dingding Zhang, Haitao Li, Xiaoxiao Shi, Yali Zheng, Limeng Chen

**Affiliations:** ^1^Department of Nephrology, State Key Laboratory of Complex Severe and Rare Diseases, Peking Union Medical College Hospital, Chinese Academy of Medical Science and Peking Union Medical College, Beijing, China; ^2^First Clinical Medical College of Norman Bethune Health Science Center, Jilin University, Changchun, China; ^3^Medical Research Center, Peking Union Medical College Hospital, Chinese Academy of Medical Sciences and Peking Union Medical College, Beijing, China; ^4^China-Japan Friendship Hospital, Jilin University, Changchun, China; ^5^Department of Nephrology, Affiliated Ningxia People's Hospital of Ningxia Medical University, Yinchuan, China

**Keywords:** uromodulin, hypertension, systolic blood pressure, diastolic blood pressure, Mendelian randomization

## Abstract

**Background:** The association of uromodulin and hypertension has been observed in clinical studies, but not proven by a causal relationship. We conducted a two-sample Mendelian randomization (MR) analysis to investigate the causal relationship between uromodulin and blood pressure.

**Methods:** We selected single nucleotide polymorphisms (SNPs) related to urinary uromodulin (uUMOD) and serum uromodulin (sUMOD) from a large Genome-Wide Association Studies (GWAS) meta-analysis study and research in PubMed. Six datasets based on the UK Biobank and the International Consortium for Blood Pressure (ICBP) served as outcomes with a large sample of hypertension (*n* = 46,188), systolic blood pressure (SBP, *n* = 1,194,020), and diastolic blood pressure (DBP, *n* = 1,194,020). The inverse variance weighted (IVW) method was performed in uUMOD MR analysis, while methods of IVW, MR-Egger, Weighted median, and Mendelian Randomization Pleiotropy RESidual Sum and Outlier (MR-PRESSO) were utilized on sUMOD MR analysis.

**Results:** MR analysis of IVM showed the odds ratio (OR) of the uUMOD to hypertension (“ukb-b-14057” and “ukb-b-14177”) is 1.04 (95% Confidence Interval (CI), 1.03-1.04, *P* < 0.001); the effect sizes of the uUMOD to SBP are 1.10 (Standard error (SE) = 0.25, *P* = 8.92E-06) and 0.03 (SE = 0.01, *P* = 2.70E-04) in “ieu-b-38” and “ukb-b-20175”, respectively. The β coefficient of the uUMOD to DBP is 0.88 (SE = 0.19, *P* = 4.38E-06) in “ieu-b-39” and 0.05 (SE = 0.01, *P* = 2.13E-10) in “ukb-b-7992”. As for the sUMOD, the OR of hypertension (“ukb-b-14057” and “ukb-b-14177”) is 1.01 (95% CI 1.01–1.02, all *P* < 0.001). The β coefficient of the SBP is 0.37 (SE = 0.07, *P* = 1.26E-07) in “ieu-b-38” and 0.01 (SE = 0.003, *P* = 1.04E-04) in “ukb-b-20175”. The sUMOD is causally associated with elevated DBP (“ieu-b-39”: β = 0.313, SE = 0.050, *P* = 3.43E-10; “ukb-b-7992”: β = 0.018, SE = 0.003, *P* = 8.41E-09).

**Conclusion:** Our results indicated that high urinary and serum uromodulin levels are potentially detrimental in elevating blood pressure, and serve as a causal risk factor for hypertension.

## Introduction

As a leading cause of cardiovascular disease, hypertension is a complex chronic clinical syndrome with multiple risk factors such as smoking (1), alcohol use (2), obesity (3), and high salt intake (4). Incidence has been rising throughout the last decades (5). At present, we have not discovered all the driving factors of hypertension.

Uromodulin, also named Tamm-Horsfall protein (THP), was first described by Carlo Rovida in 1873. It is produced by the cells in the thick ascending limb (TAL) and the distal convoluted tubule (DCT) with daily secretion of 50–150 mg in urine (6). Uromodulin is physiologically secreted into the renal interstitium, enters the blood to form serum uromodulin (sUMOD), with a level <0.001 of the level of urinary uromodulin (uUMOD) (7). uUMOD plays a crucial role in various biochemical processes, such as protection against urinary tract infection, immunomodulation, and regulating water and salt balance (8). sUMOD was significantly associated with many diseases, such as impaired glucose metabolism, kidney function, and risk for kidney allograft failure (9–11). The association of uUMOD level and salt-sensitive hypertension was observed, but the causal effect of uUMOD on hypertension has not been confirmed (12). Since traditional observational studies might be biased by many underlying confounders such as lifestyles and socioeconomic status (5), the cost of a large randomized controlled trial (RCT), or cohort studies is extremely expensive; as such few studies have focused on exploring the causal relationship between uromodulin and hypertension. Therefore the causal effect of uromodulin on hypertension requires a new strategy in order to be investigated.

“Mendelian randomization” (MR) is an emerging research method that can simulate randomized controlled trials using genetic variants (usually single nucleotide polymorphisms, SNPs) as instrumental variables. Because the gene is allocated randomly at conception (13), MR was designed as a natural randomization method that could minimize the effects of confounders. Nowadays, the MR method has been widely applied to estimate the causal effect of exposure on outcome, and successfully confirmed that lower low-density lipoprotein (LDL) cholesterol contributed to fewer cardiovascular events (14). Recently, many studies have tried to disentangle the risk factors for hypertension by MR methods. Besides some traditional risk factors for hypertension such as body weight index (BMI), adiposity, dietary dairy consumption, smoking, and alcohol intake, it also disclosed some further potential risk factors; namely uric acid, vitamin D levels, gamma-glutamyl transferase, total bilirubin, glycated hemoglobin, beta-2-microglobulin, and apolipoprotein E (15).

In this study, we tried to use two-sample MR methods to unveil the causal effect of uromodulin on hypertension, systolic blood pressure (SBP), and diastolic blood pressure (DBP) using increasingly available public genome-wide association studies (GWAS) datasets.

## Methods and Materials

### Study Design

**Figure 1:**
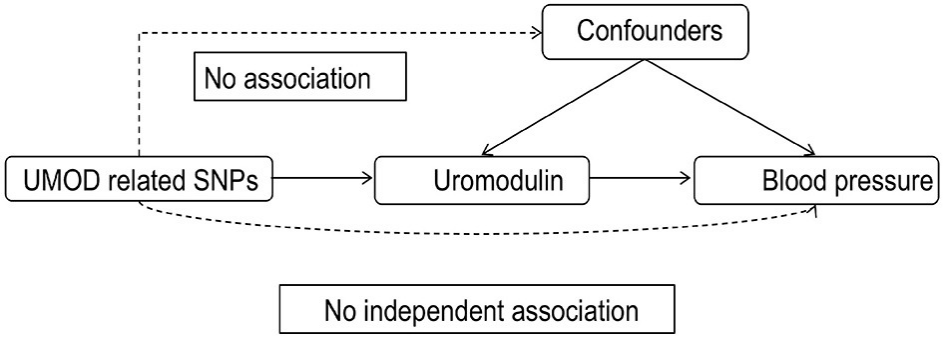
Directed acyclic graph composed of the genetic instrument (UMOD-related SNPs), exposure (UMOD), and outcome (blood pressure). SNPs, Single nucleotide polymorphisms; UMOD, Uromodulin.

MR analysis is based on three assumptions: (1) The instrumental variable (IV) is closely associated with the exposure. (2) The IV is not associated with any potential confounders. (3) The IV can only influence the outcome via the exposure, and not by any other ways. We constructed a directed acyclic graph by using genetic instruments (UMOD-related SNPs), exposures (serum uromodulin and urinary uromodulin), and outcomes (hypertension, diastolic blood pressure, and systolic blood pressure, Figure 1).

### IVs Selection

We initially extracted all five SNPs associated with uUMOD levels from the largest GWAS meta-analysis of uUMOD (16). This study was a fixed-effects meta-analysis combining results of 10,884 participants of European descent, consisting of three genetic isolates and three urban cohorts. The details of the SNPs are in Supplementary Table 1. We selected IVs with *P* ≤ 5 × 10^−8^, minor allele frequency (MAF) >0.01, and low linkage disequilibrium (LD) (r2 < 0.1). Finally, two SNPs (rs12917707 and rs4494548) were valid for further MR analysis of uUMOD.

sUMOD-related SNPs were obtained from 4,147 participants in the Outcome Reduction with Initial Glargine Intervention (ORIGIN) trial (17), which selected SNPs within 300 kb of the UMOD gene significantly associated with sUMOD, and pruned the SNPs for LD at a threshold of r2 > 0.1 using 1,000 Genomes data (Europeans). Sixteen SNPs were selected as the IVs in the MR analysis of sUMOD, and rs12446494 was excluded for high LD to rs12917707 (r2 = 0.16) (Supplementary Table 2).

### Outcome Data Sources

**Figure 2:**
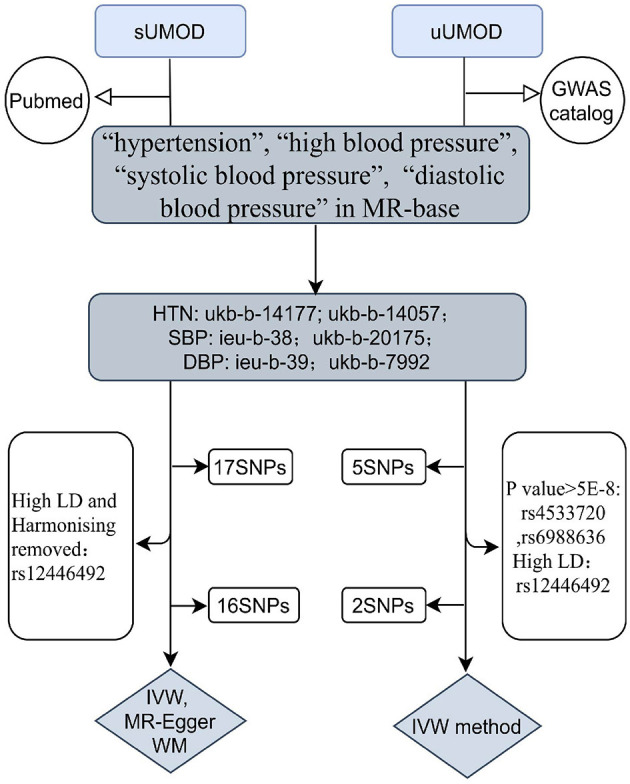
The flow chart of the study design. For instrumental variants (IVs) of the uUMOD, we removed two SNPs (rs4533720 and rs6988636) because of their *p*-value (>5e-8), and one SNP (rs12446492) due to its high linkage disequilibrium (LD) to rs12917707 (r20 = 0.16). For IVs of the sUMOD, rs12446492 was excluded for the same reason as uUMOD being palindromic with intermediate allele frequencies. uUMOD, Urinary uromodulin; sUMOD, Serum uromodulin; SBP, Systolic blood pressure; DBP, Diastolic blood pressure; HTN, Hypertension; IVW, Inverse variance weighted; WM, Weighted median; MR-Egger, Mendelian randomization-Egger; SNPs, Single Nucleotide Polymorphisms.

We extracted the outcome data (blood pressure) from the MR-base database (18) (https://gwas.mrcieu.ac.uk/), which is a curated database including a summary originated from 1,094 GWASs involving 889 traits of physiological characteristics and disease phenotypes. We searched the traits “hypertension”, “high blood pressure”, “systolic blood pressure”, and “diastolic blood pressure” as keywords, filtered by the European population up to 2020 in the MR-base database. We chose the largest sample size study with available data in the different consortium as outcomes (Figure 2). Two summary datasets with IDs “ukb-b-14057” (non-cancer illness code, self-reported: Hypertension) and “ukb-b-14177” (vascular/heart problems diagnosed by doctor: High blood pressure) were selected as the outcome of hypertension and high blood pressure. They were originated from the MRC Integrative Epidemiology Unit (MRC-IEU) consortium (http://www.bristol.ac.uk/integrative-epidemiology/) based on the UK Biobank, which is a large and detailed genotyped biobank that has globally recruited over 500,000 participants (aged 40–69 years) between 2006 and 2010 (19). The “ukb-b-14057” ID contains 46,293 people while “ukb-b-14177” includes 46,188 participants. We selected two summary datasets “ukb-b-20175” (systolic blood pressure, automated reading) and “ieu-b-38” (systolic blood pressure) as the outcome of SBP. The IDs “ieu-b-39” (diastolic blood pressure) and “ukb-b-7992” (diastolic blood pressure, automated reading) were selected as the datasets of the DBP. The “ieu-b-38” (systolic blood pressure) and “ieu-b-39” IDs (diastolic blood pressure) included summary level data based on the International Consortium for Blood Pressure (ICBP) (20) (www.ncbi.nlm.nih.gov/projects/gap/cgibin/study.cgi?study_id=phs000585.v1.p1), which is a multi-stage GWAS study of systolic and diastolic blood pressure in 200,000 individuals of European descent (21, 22). While “ukb-b-20175” and “ukb-b-7992” included summary data of the UK biobank (Supplementary Tables 3, 4).

### MR Analysis

In two-sample MR, it is necessary to ensure that the effect allele of IVs in exposure and outcome between different databases correspond to the same allele. Thus, we tried to infer the forward strand alleles using allele frequency information to harmonize the data and discarded ambiguous IVs or not inferable palindromic ones.

For IVs with more than three SNPs, we performed MR analysis through several robust analytical methods based on different assumptions of two-sample MR analysis; namely inverse variance weighted (IVW), MR-Egger, and weighted median (WM). The IVW method utilizes a meta-analysis approach to pool Wald ratios for each SNP (i.e., the β coefficient of the SNP for UMOD is divided by the β coefficient of the SNP for outcomes) to get the combined estimates of the effect of uromodulin on outcomes (hypertension, DBP, SBP) (23). MR-Egger regression makes a weighted linear regression of the outcome coefficients on the exposure coefficients. It can provide unbiased estimates even when all genetic variants are invalid (24). The WM method calculates the median of the empirical distribution of MR association estimates weighted for their precision and offers consistent estimates. For IVs with less than three SNPs, we performed MR analysis by the IVW method.

We performed Cochran's Q statistic to assess heterogeneity between individual genetic variants in the IVW method. A random-effects model was used when the heterogeneity was high (25). We then conducted scatter plots and the leave-one-out method to evaluate the robustness of these findings. To confirm the influential outliers and horizontal pleiotropy, we adopted MR-PRESSO (Mendelian Randomization Pleiotropy RESidual Sum and Outlier) to detect and correct for potential outliers (*P* < 0.05). We also used MR-PRESSO to test the significant differences in the causal estimates before and after correction for outliers (26) and the intercept of MR-Egger to further test the horizontal pleiotropy (*P* < 0.05). The analysis was performed by packages “Two Sample MR” and “MR-PRESSO” in R 4.0.2 software.

### Power Calculation and Weak Instrument Bias

We used the F statistic to evaluate the strength of the association between SNP and exposure. The formula to calculate the F statistic is F = $\frac{N-k-1}{k}\times \frac{R^{2}}{1-R^{2}}\;$(27). Where N represents the sample size, *k* is the number of SNPs. The variance (*R*^2^) represents the phenotype variance induced by the SNPs. When *R*^2^ is not available, we use the formula *R*^2^ = 2 × MAF × (1–MAF) × beta^2^ (where beta represents the effect value of the genetic variant in the exposure and MAF represents the effect allele frequency) (28). When the F statistic is >10, it reveals a strong correlation between SNP and exposure with sufficient statistical power. Combined F statistics were also conducted to further assess weak instrument bias. We recalculated the power using a web-based application (https://sb452.shinyapps.io/power/) (29).

## Results

### uUMOD MR Analysis

For the outcome of hypertension, we observed that the elevated urinary uromodulin level could increase the risk of hypertension in dataset “ukb-b-14057” (Oodds ratio (OR) = 1.036, 95% CI, 1.029–1.043, *P* = 3.07E−26) and “ukb-b-14177” (OR = 1.036, 95% CI, 1.030–1.044, *P* = 3.29E−26) (Table 1). In the MR analysis of SBP, uUMOD is significantly causally associated with the SBP in “ieu-b-38” (β = 1.100, standard error (SE) = 0.25, *P* = 8.92E-06) and “ukb-b-20175” (β = 0.03, SE = 0.01, *P* =2.70E-04). The causal relationship between uUMOD and DBP was significant, as the β coefficient of “ieu-b-39” is 0.88 (SE = 0.19, *P* = 4.38E−06) and 0.05 for “ukb-b-7992” (SE = 0.01, *P* = 2.13E−10).

**Table 1:** The outcomes of two-sample Mendelian randomization.

**Trait**	**Id.outcome**	**Method**	**sUMOD**			**uUMOD**		
			**BETA**	**SE**	***P*-value**	**BETA**	**SE**	***P*-value**
Hypertension	ukb-b-14057	IVW	0.013084182	0.001551856	8.06E-21	0.035563214	0.0033559	3.07E-26
		MR-Egger	0.015399292	0.002209318	3.18E-09			
		WM	0.014523675	0.00330377	3.67E-04			
High blood pressure	ukb-b-14177	IVW	0.01307694	0.002270396	8.42E-09	0.036032977	0.003402303	3.29E-26
		MR-Egger	0.016328943	0.003299669	2.14E-04			
		WM	0.01470498	0.001546514	1.93E-21			
Systolic blood pressure	ieu-b-38	IVW	0.370736243	0.070147745	1.26E-07	1.09960196	0.247554764	8.92E-06
		MR-Egger	0.579574617	0.074086021	2.85E-06			
		WM	0.496799596	0.055636561	4.28E-19			
	ukb-b-20175	IVW	0.010683065	0.002753179	1.04E-04	0.028258738	0.007757293	2.70E-04
		MR-Egger	0.014238981	0.004101904	3.74E-03			
		WM	0.012356676	0.003208704	1.18E-04			
Diastolic blood pressure	ieu-b-39	IVW	0.313093826	0.049871445	3.43E-10	0.881848889	0.192013206	4.38E-06
		MR-Egger	0.441395172	0.059915509	5.45E-06			
		WM	0.365472086	0.034446225	2.68E-26			
	ukb-b-7992	IVW	0.017532188	0.003043809	8.41E-09	0.049155426	0.007738922	2.13E-10
		MR-Egger	0.023557937	0.004161065	5.87E-05			
		WM	0.019209604	0.003139275	9.41E-10			

*(MR) analysis with sUMOD and uUMOD. All methods of the MR analysis outcomes are significant (p < 0.05)*.

*IVW, Inverse variance weighted; WM, Weighted median; SE, Standard error; uUMOD, Urinary uromodulin; sUMOD, Serum uromodulin; SNP, Single nucleotide polymorphisms; MR, Mendelian randomization*.

### sUMOD MR Analysis

**Figure 3:**
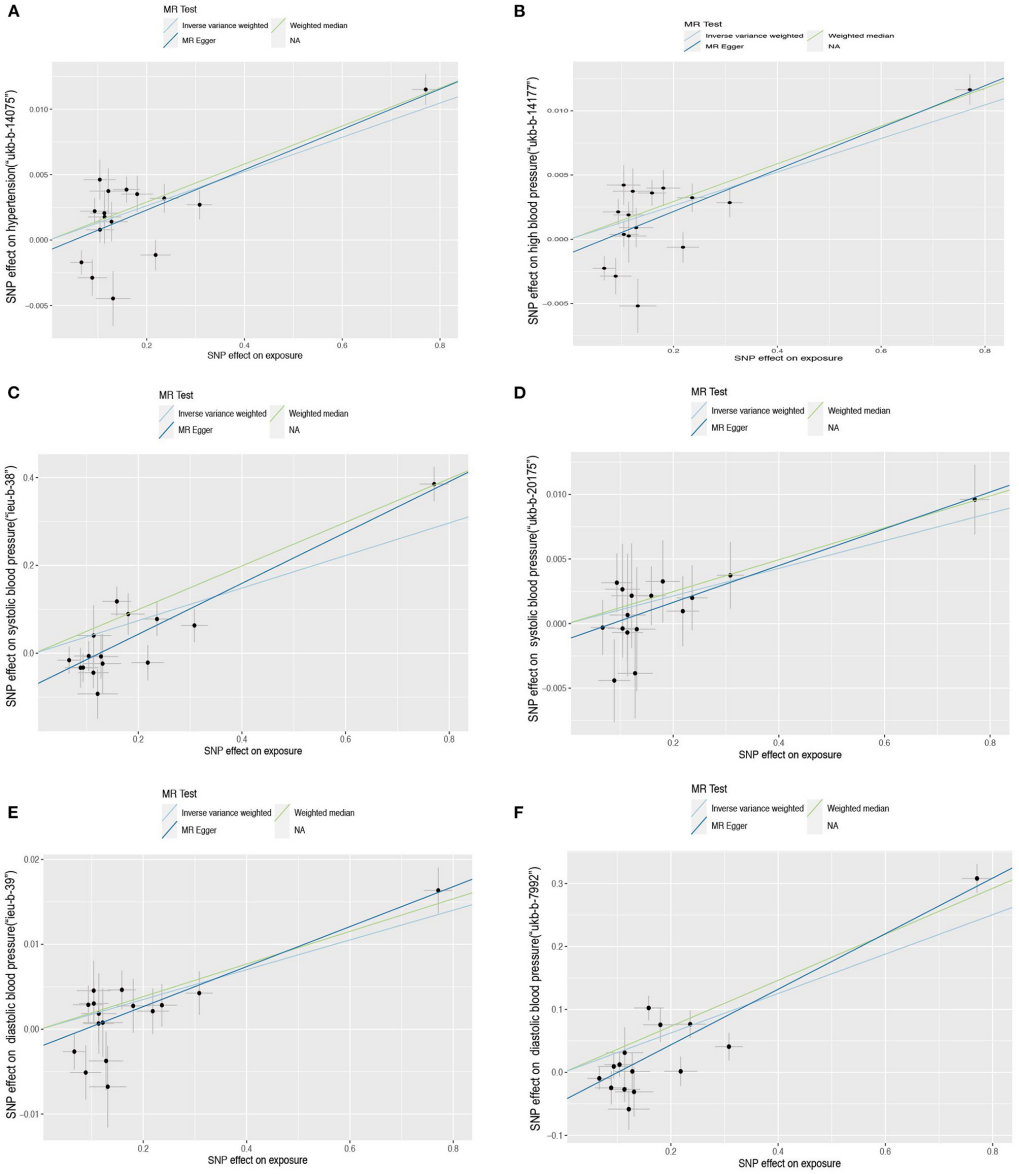
Scatter plot of the Mendelian randomization (MR) outcome. Relationship between the effect size estimates on sUMOD (x-axis) and the effect size estimates on outcomes (y-axis): Hypertension **(A,B)**, systolic blood pressure **(C,D)**, diastolic blood pressure **(E,F)**. The slope of fitted lines represents the estimated causal effect of sUMOD obtained using the inverse variance weighted (IVW), MR-Egger, and weighted median (WM). Hypertension: **(A)** (ukb-b-14177), **(B)** (ukb-b-14-57); systolic blood pressure: **(C)** (ieu-b-38), **(D)** (ukb-b-20175); diastolic blood pressure: **(E)** (ieu-b-39), **(F)** (ukb-b-7992).

**Figure 4:**
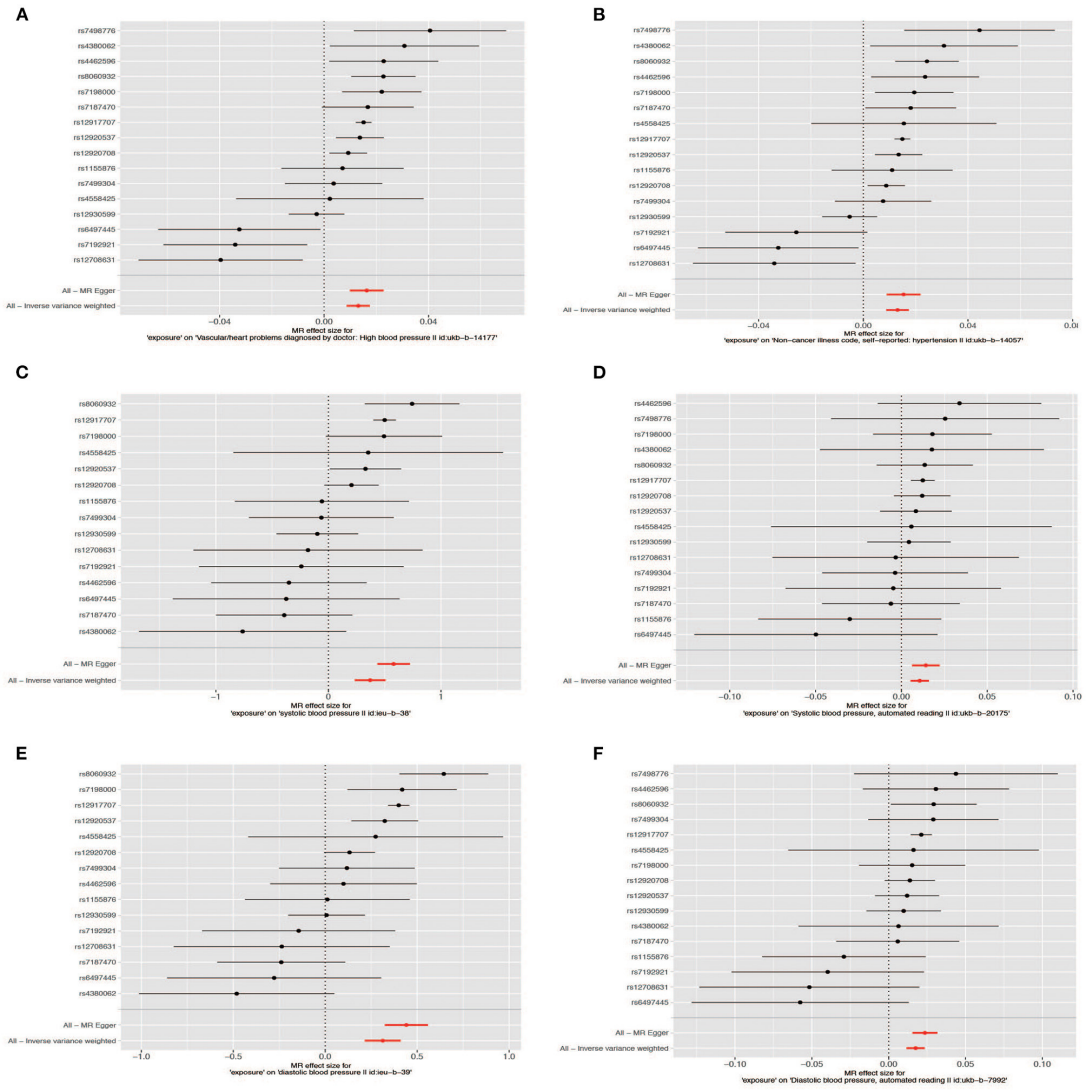
Forest plot of the Mendelian randomization (MR) outcome. Hypertension: **(A)** (ukb-b-14177), **(B)** (ukb-b-14-57); systolic blood pressure: **(C)** (ieu-b-38), **(D)** (ukb-b-20175); diastolic blood pressure: **(E)** (ieu-b-39), **(F)** (ukb-b-7992).

The effect of sUMOD on hypertension is consistent with uUMOD as the IVW outcome of “ukb-b-14057” and “ukb-b-14177” is the same (OR = 1.013, 95% CI 1.009-1.0018, all *P* < 0.001). For the outcome of SBP, the β coefficient of IVW in “ieu-b-38” is 0.371 (SE = 0.070, *P* = 1.26E−07) and 0.011 in “ukb-b-20175” (SE = 0.003, *P* = 1.04E−04). In the MR analysis, the IVW outcome of the DBP in “ieu-b-39” and “ukb-b-7992” are both significant (β = 0.313 with SE = 0.050 and β = 0.018 with SE = 0.003, respectively, all *P* < 0.001). All outcomes of three-method MR analysis are shown in Table 1 and Figures 3, 4.

### Heterogeneity and Pleiotropy Test

The Scatter plot shows the distribution of the single SNP's effect on the outcome (Figure 3). High heterogeneity was found in hypertension (“ukb-b-14177”), high blood pressure (“ukb-b-14057”), SBP (“ieu-b-38”), and DBP (“ieu-b-39”); while SBP (“ukb-b-20175”) and DBP (“ukb-b-7992”) possess low heterogeneity. The leave-one-out method suggested the outcome is robust except for SBP (“ukb-b-20175” and “ieu-b-38”) (Supplementary Figure 1). The *p*-value of the MR-Egger intercept is more than 0.05 in hypertension, high blood pressure, and SBP (the dataset “ukb-20175”), indicating no evidence of genetic pleiotropy, while <0.05 in DBP and part of SBP (the dataset “ieu-b-38”). Further horizontal pleiotropy testing with MR-PRESSO showed there are no outliers in DBP (“ukb-b-7992”) and SBP (“ukb-20175”). In the outcome of hypertension (“ukb-b-14177”) and high blood pressure (“ukb-b-14057”), though outliers existed, the corrected outcomes are consistent with the global rate. Rs12917707 and rs12930599 are outliers in the DBP (“ieu-b-39”) with the corrected outcome (β = 0.190, *P* = 2.37E−02). The MR-PRESSO outcome in SBP (”ieu-b-38“) is not significant when rs12917707 was excluded as an outlier (Table 2).

**Table 2:** Outcomes of Mendelian Randomization Pleiotropy RESidual Sum and Outlier (MR-PRESSO).

**Datasets ID**	**Outliers**	**Corrected beta**	***P* value**
ukb-b-14177	rs12708631,rs12917707 rs12930599,rs7192921	0.014	6.93E−04
ukb-b-14057	rs12708631,rs12917707 rs2930599,rs7192921	0.012	4.00E−03
ieu-b-39	rs12917707,rs12930599	0.19	2.37E−02
ukb-b-7992	NA	NA	NA
ieu-b-38	rs12917707	0.148	0.13
ukb-b-20175	NA	NA	NA

### Power Calculation

In the uUMOD MR analysis, the F statistics of rs12917707 and rs4494548 are 169.58 and 35.80, respectively. The mean F statistic of the sUMOD-related SNPs is 78.28. The high F statistic (empirically > 10) indicated a strong association between SNPs and urinary uromodulin and less weak instrument bias. The power of our MR analysis in different pairs was over 90% at an alpha rate of 5%, except the dataset “ukb-b-20175” (systolic blood pressure) which was 75.2% (Supplementary Table 5).

## Discussion

Our MR analysis unveiled the causal effect of both uUMOD and sUMOD on blood pressure by integrating publicly available GWAS datasets. High sUMOD and uUMOD could contribute to the risk of hypertension (the biggest OR is 1.036, 95% CI, 1.030–1.044). Both sUMOD and uUMOD are causally associated with both SBP (the largest causal estimate being a 0.10 mmHg per unit change in uromodulin) and DBP (the largest causal estimate being a 0.88 mmHg per unit change in uromodulin).

To our limited knowledge, this is the first study designed to research the causal association of both serum and urinary uromodulin and hypertension by MR methods with potential confounders removed by genetic variants. One abstract using MR analysis to reveal the causal association of uUMOD of DBP and SBP only included the “ieu-b-39” and “ieu-b-38” datasets (30). We screened all the summary studies of DBP and SBP on the MR-base up to 2020 and added two datasets. Besides, we further assessed the causal effect of uromodulin on hypertension, utilizing the open data showing the association between SNPs and sUMOD.

We adopted three methods based on different assumptions to ensure our outcome. MR-Egger relies on the assumption that the SNP should affect the risk of the outcome through the exposure, not via other risk factors; namely Instrument Strength Independent of Direct Effect (InSIDE). The WM method does not require InSIDE to be taken into account. This method provides valid estimates when at least 50% of the weight comes from valid variants. It can improve the power of causal effect detection and decrease type I error with distinct superiorities over MR-Egger (31). MR-PRESSO enhances the detection of outliers by rigorously exploring whether the findings were biased due to pleiotropy. Although we cannot entirely rule out pleiotropy, we observed a consistent outcome between uromodulin levels and blood pressure in conventional MR analysis. Our results based on different methods and datasets strengthen the theoretical support for further well-designed prospective randomized clinical trials to verify the causal association of uromodulin and hypertension, larger than those that came before them. Furthermore, our results may further suggest that uromodulin might serve as a new therapeutic target for hypertension management.

The correlation between uromodulin and hypertension was first disclosed in 1998. Duława, J. reported that compared with healthy control, uUMOD excretion was significantly higher in hypertensive individuals (32); and could be normalized by angiotensin converting enzyme inhibitors (ACEI) (33). It is consistent in pre-eclampsia patients (34). RNA-seq data of wild-type (WT) mice treated by a high salt diet showed a significant upregulation of heat-shock proteins Hspa1b (Hsp70) and blood pressure that were both abolished in UMOD knockout mice (35–37). It indicated the potential causal relationship between UMOD and hypertension, but was difficult to confirm in a population study. Our study has shown that UMOD was a causal factor of hypertension by utilizing the Mendelian randomization method.

The underlying mechanism of uromodulin influencing blood pressure is due to its regulation of the ion channel's activity in TAL and DCT, including the renal outer medullary potassium channel (ROMK), epithelial sodium channel (ENaC), Na+-K+-2Cl- cotransporter (NKCC2), and Na+-Cl– cotransporter (NCC). Animal studies proved that UMOD could upregulate the ROMK and ENaC expression in TAL (38) and lead to salt-sensitive hypertension. UMOD knockout mice presented significantly lower systolic blood pressure compared to WT mice under basal conditions (39, 40). UMOD transgenic mice with increased expression and secretion of uromodulin showed higher BP and a significant increase of NKCC2 phosphorylation at activating sites (Thr96 and Thr101). In an *in vitro* study, co-expression of uromodulin in renal cells induced an obvious increase of NKCC2 phosphorylation and its activity (12). Uromodulin also facilitated NCC phosphorylation which was possible via SPS1-related proline/alanine-rich kinase/oxidative stress response kinase 1 (SPAK-OSR1) modulation (41). The upregulation activity of both NKCC2 and NCC contribute to NaCl reabsorption and retention, leading to salt-sensitive hypertension (42, 43).

Our study had some limitations. First, since all the data came from people of European origin, the results were not representative of a truly random population sample nor applicable to other ethnicities. In the uUMOD MR, we only included two SNPs, which meant we could not conduct MR-Egger, a median-based estimator, model-based estimators, and other analysis methods to examine the horizontal pleiotropy. Second, there was a likely overlap of uUMOD (population from the Framingham Heart Study: 24%) and ICBP (0.3%). However, the overlapping degree is small in ICBP and we included the UK Biobank to confirm the outcome. We assessed the effect of the overlap in the online app (https://sb452.shinyapps.io/overlap/), it showed that when the overlap is below 30%, the bias is <0.018 (44). Third, due to the presence of strong instruments, we consider this overlap not to introduce significant bias (45). Fourth, we could not perform the bidirectional Mendelian randomization owing to a lack of effect size data on hypertension-related SNPs in the exposure population. Fifth, due to the lack of individual data, all MR methods tested only the linear effect of uromodulin on blood pressure, and could not exclude a modest or non-linear effect. Finally, as this was an MR analysis, we also could not overcome general limitations such as the possibility of population stratification, the pleiotropy of SNPs, and canalization (15).

## Conclusion

In conclusion, our study based on open datasets suggests a potentially detrimental impact of high levels of uromodulin on the development of hypertension; which is the first time this has been shown to be consistent with the observational study and basic experimental study.

## Data Availability Statement

The datasets presented in this study can be found in online repositories. The names of the repository/repositories and accession number(s) can be found in the article/Supplementary Material.

## Author Contributions

RY conceived and designed the study, performed the study, analyzed the data, wrote the paper, and prepared figures and/or tables. LC performed the MR analysis, analyzed the data, and reviewed drafts of the paper. LX and DZ analyzed the data, prepared figures and tables, and reviewed drafts of the paper. HL performed the study, analyzed the data, and prepared figures and tables. XS designed the study and reviewed drafts of the paper. YZ analyzed the data and reviewed drafts of the paper. LC conceived and designed the study, wrote the paper, and reviewed drafts of the paper. All authors contributed to the article and approved the submitted version.

## Conflict of Interest

The authors declare that the research was conducted in the absence of any commercial or financial relationships that could be construed as a potential conflict of interest.

## Publisher's Note

All claims expressed in this article are solely those of the authors and do not necessarily represent those of their affiliated organizations, or those of the publisher, the editors and the reviewers. Any product that may be evaluated in this article, or claim that may be made by its manufacturer, is not guaranteed or endorsed by the publisher.
