# Supplementary material for: High Level of Uromodulin Increases the Risk of Hypertension: A Mendelian Randomization Study
Source: Front Cardiovasc Med. 2021 Sep 1;8:736001. doi: 10.3389/fcvm.2021.736001 (PMC8440862; doi:10.3389/fcvm.2021.736001)

Supplementary materials

Table S1. Details of the UMOD related SNPs. uUMOD related SNPs were derived from the Genome-Wide Association Studies (GWAS) meta-analysis in GWAScatalog. A total of seventeen SNPs associated with uUMOD were from an article published in the Journal of American Society of Nephrology.
EA, Effect allele; *OA*, Other allele; *EAF,* Effect Allele Frequency; *BETA,* Effect size of SNP on uromodulin; *SE*, Standard Error; *uUMOD*, urinary uromodulin; *sUMOD*, serum uromodulin; *SNPs*, Single Nucleotide Polymorphisms

Table S2. The variance (R2) of UMOD-blood pressure pairs. *SNP*, Single Nucleotide Polymorphism; *UMOD*, uromodulin

Table S3. Studies of hypertension with data available from the Mendelian randomization (MR)-base.
Outcomes of the "hypertension" and “high blood pressure" searched in MR-base were shown in the table. We selected the ukb-b-14057 and ukb-b-14177 since their largest Ncase and the sample size.

Table S4. Studies of systolic blood pressure and diastolic blood pressure from the Mendelian randomization (MR)-base. We selected ieu-b-38 and ukb-b-20175 as the outcome of systolic blood pressure, while ieu-b-39 and ukb-b-7992 as the outcome of diastolic blood pressure.
ICBP, International Consortium for Blood Pressure; *NSNPs*, Number of Single Nucleotide Polymorphisms

Table S5. Detailed power of each pair. *UMOD*, uromodulin; *SBP,* Systolic blood pressure; *DBP*, diastolic blood pressure

Figure S1. Leave-one-out method of the Mendelian randomization(MR) outcome.
Hypertension: A (ukb-b-14177), B (ukb-b-14-57); Systolic blood pressure: C (ieu-b-38), D (ukb-b-20175); Diastolic blood pressure: E (ieu-b-39), F (ukb-b-7992)

Table S1.Details of the UMOD related SNPs. uUMOD related SNPs were derived from the Genome-Wide Association Studies(GWAS) meta-analysis in GWAScatalog. A total of seventeen SNPs associated with uUMOD were from an article published in the Journal of American Society of Nephrology.

Exposure	SNP	EA	OA	EAF	P value	BETA	SE	R^2
uUMOD	rs12917707	T	G	0.1800	7.85E-73	-0.3200	0.0200	0.0120
	rs4494548	A	G	0.1300	3.90E-15	0.1700	0.0200	0.0080
	rs12446492	A	T	0.4500	5.60E-27	-0.1500	0.0100	0.0111
	rs4533720	A	G	0.5300	3.50E-07	0.0700	0.0100	0.0024
	rs6988636	T	C	0.9200	9.47E-07	-0.1300	0.0300	0.0025
sUMOD	rs12917707	T	G	0.1510	8.35E-154	-0.7709	0.0280	0.1524
	rs1155876	C	T	0.0840	0.00011	-0.1280	0.0331	0.0025
	rs12446492	A	T	0.3890	1.05E-43	-0.3293	0.0235	0.0515
	rs12708631	A	T	0.0750	0.000241	-0.1312	0.0357	0.0024
	rs12920537	G	A	0.310	1.16E-15	-0.2360	0.0293	0.0238
	rs12920708	C	A	0.230	1.50E-32	-0.3083	0.0257	0.0337
	rs12930599	A	G	0.2190	1.77E-12	-0.2183	0.0309	0.0163
	rs4380062	T	C	0.0860	0.00172	-0.1215	0.0387	0.0023
	rs4462596	G	A	0.2610	0.00159	-0.0935	0.0296	0.0034
	rs4558425	A	G	0.0580	0.000876	-0.1138	0.0342	0.0014
	rs6497445	C	T	0.1330	0.00331	0.0886	0.0302	0.0018
	rs7187470	A	G	0.4510	0.00011	0.1134	0.0293	0.0064
	rs7192921	G	T	0.4060	0.00274	0.0665	0.0222	0.0021
	rs7198000	A	G	0.1240	2.82E-08	0.1804	0.0324	0.0071
	rs7498776	C	T	0.0800	0.0014	0.1040	0.0325	0.0016
	rs7499304	T	G	0.2880	0.0002	-0.1043	0.0284	0.0045
	rs8060932	G	A	0.3150	7.23E-09	0.1586	0.0274	0.0109

EA, Effect allele; *OA*, Other allele; *EAF,* Effect Allele Frequency; *BETA,* Effect size of SNP on uromodulin; *SE*, Standard Error; *uUMOD*, urinary uromodulin; *sUMOD*, serum uromodulin; *SNPs*, Single Nucleotide Polymorphisms

Table S2. The variance (R2) of UMOD-blood pressure pairs.

Exposure	SNP	Hypertension		Systolic blood pressure		Diastolic blood pressure	
		ukb-b-14057	ukb-b-14177	ieu-b-38	ukb-b-20175	ieu-b-39	ukb-b-7992
sUMOD	rs1155876	3.54E-07	1.46E-07	9.98E-06	2.65E-06	4.09E-07	2.50E-06
	rs12446492	1.57E-05	1.59E-05	0.021	1.87E-05	0.0147	5.27E-05
	rs12708631	1.90E-06	2.58E-06	6.05E-05	1.82E-08	0.0001	4.38E-06
	rs12917707	3.92E-05	4.02E-05	0.0438	2.73E-05	0.028	7.91E-05
	rs12920537	4.00E-06	4.12E-06	0.0024	1.56E-06	0.0023	3.15E-06
	rs12920708	2.47E-06	2.76E-06	0.0014	4.73E-06	0.0006	6.13E-06
	rs12930599	4.42E-07	1.32E-07	0.0002	3.13E-07	8.75E-07	1.54E-06
	rs4380062	1.96E-06	1.95E-06	0.0013	6.51E-07	0.0005	8.44E-08
	rs4462596	2.06E-06	1.93E-06	0.0005	4.24E-06	3.65E-05	3.50E-06
	rs4558425	2.96E-07	6.00E-09	0.0002	4.16E-08	9.28E-05	3.22E-07
	rs6497445	1.76E-06	1.75E-06	0.0002	4.13E-06	0.0001	5.52E-06
	rs7187470	2.03E-06	1.74E-06	0.001	2.31E-07	0.0004	2.16E-07
	rs7192921	1.38E-06	2.45E-06	0.0001	4.71E-08	4.41E-05	3.33E-06
	rs7198000	2.64E-06	3.40E-06	0.0017	2.28E-06	0.0012	1.62E-06
	rs7499304	2.64E-07	6.14E-08	1.78E-05	6.15E-08	6.38E-05	3.88E-06
	rs8060932	6.48E-06	5.65E-06	0.0060	2.02E-06	0.0045	9.39E-06
	rs7498776	3.71E-06	3.10E-06	NA	1.22E-06	NA	3.61E-06
uUMOD	rs12917707	3.92E-05	4.02E-05	0.0438	2.73E-05	0.0280	7.91E-05
	rs4494548	6.13E-06	6.31E-06	0.0014	1.90E-06	0.0010	8.08E-06

SNP, Single Nucleotide Polymorphism; *UMOD*, uromodulin

Table S3. Studies of hypertension with data available from the Mendelian randomization (MR)-base.
Outcomes of the "hypertension" and “high blood pressure" searched in MR-base were shown in the table. We selected the ukb-b-14057 and ukb-b-14177 since their largest Ncase and the sample size.

Trait	Id.outcome	Detailed Trait	Year	Author	Consortium	Ncase	Ncontrol	Sample size	NSNPs
Hypertension	ukb-a-61	Non-cancer illness code self-reported: hypertension	2017	Neale	Neale Lab	87690	249469	337,159	10894596
	ukb-a-531	Diagnoses-main ICD10:I10 Essential (primary) hypertension	2017	Neale	Neale Lab	500	336699	337,199	10894596
	ukb-b-14057	Non-cancer illness code, self-reported: hypertension	2018	Ben Elsworth	MRC-IEU	119731	343202	462,933	9851867
	ukb-b-7582	Non-cancer illness code, self-reported: essential hypertension	2018	Ben Elsworth	MRC-IEU	2095	460838	462,933	9851867
	ukb-b-12493	Diagnoses - secondary ICD10: I10 Essential (primary) hypertension	2018	Ben Elsworth	MRC-IEU	54358	408652	463,010	9851867
	ukb-d-I9_HYPTENS	Hypertension	2018	Neale	NA	1237	159957	361,194	9646741
High blood pressure	ukb-a-437	Vascular/heart problems diagnosed by doctor: High blood pressure	2017	Neale	Neale Lab	NA	NA	336683	10894596
	ukb-b-14177	Vascular/heart problems diagnosed by doctor: High blood pressure	2018	Ben Elsworth	MRC-IEU	NA	NA	461880	9851867

MR, Mendelian randomization; *NSNPs*, Number of Single Nucleotide Polymorphisms

Table S4. Studies of systolic blood pressure and diastolic blood pressure from the Mendelian randomization (MR)-base. We selected ieu-b-38 and ukb-b-20175 as the outcome of systolic blood pressure, while ieu-b-39 and ukb-b-7992 as the outcome of diastolic blood pressure.

Trait	Id.outcome	Detailed Trait	Year	Author	Consortium	Sample size	NSNPs
Systolic blood pressure	ieu-b-38	Systolic blood pressure	2018	Evangelou, E	ICBP	757601	7088083
	ukb-a-360	Systolic blood pressure automated reading	2017	Neale	Neale Lab	317754	10894596
	ukb-b-20175	Systolic blood pressure, automated reading	2018	Ben Elsworth	MRC-IEU	436419	9851867
	ukb-b-6503	Systolic blood pressure, manual reading	2018	Ben Elsworth	MRC-IEU	39749	9851867
Diastolic blood pressure	ieu-b-39	Diastolic blood pressure	2018	Evangelou, E	ICBP	757601	7160619
	ukb-a-359	Diastolic blood pressure automated reading	2017	Neale	Neale Lab	317756	10894596
	ukb-b-7992	Diastolic blood pressure, automated reading	2018	Ben Elsworth	MRC-IEU	436424	9851867
	ukb-b-18240	Diastolic blood pressure, manual reading	2018	Ben Elsworth	MRC-IEU	39749	9851867

ICBP, International Consortium for Blood Pressure; *NSNPs*, Number of Single Nucleotide Polymorphisms

Table S5. Detailed power of each pair.

Exposure	Outcome	outcome datasets ID	Power
sUMOD	Hypertension	ukb-b-14057	99.60%
	High blood pressure	ukb-b-14177	99.60%
	SBP	ieu-b-38	100%
		ukb-b-20175	93.20%
	DBP	ieu-b-39	100%
		ukb-b-7992	100%
uUMOD	hypertension	ukb-b-14057	92.80%
	high blood pressure	ukb-b-14177	93.40%
	SBP	ieu-b-38	100%
		ukb-b-20175	75.20%
	DBP	ieu-b-39	100%
		ukb-b-7992	99.60%

UMOD, uromodulin; *SBP,* Systolic blood pressure; *DBP*, diastolic blood pressure

Figure S1. Leave-one-out method of the Mendelian randomization(MR) outcome. Hypertension: A(ukb-b-14177), B(ukb-b-14-57); Systolic blood pressure: C(ieu-b-38), D(ukb-b-20175);Diastolic blood pressure: E(ieu-b-39), F(ukb-b-7992)

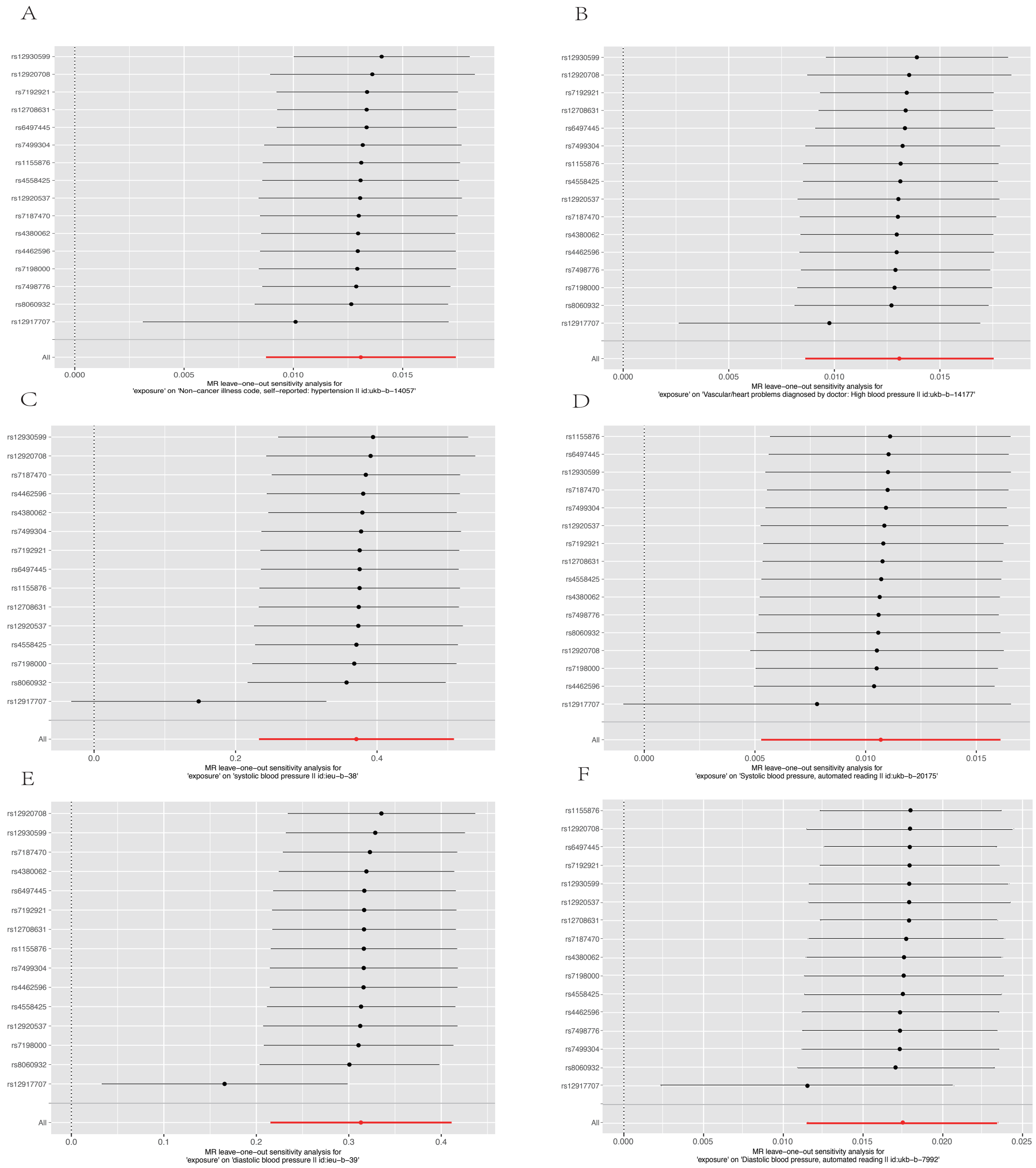

Supplement: Supplementary file 1 [file Table_1.pdf]
